# Bioactive Compounds and Antioxidant Capacity of Pulp, Peel and Seeds from Jeriva (*Syagrus romanzoffiana*)

**DOI:** 10.3390/antiox13060711

**Published:** 2024-06-12

**Authors:** Beatriz C. B. S. Mello, Angelika Malarski, Volker Böhm

**Affiliations:** 1 Center for Natural Sciences, Federal University of São Carlos, Campus Lagoa do Sino, Buri 18290-000, Brazil; 2 Institute of Nutritional Sciences, Friedrich Schiller University Jena, 07743 Jena, Germany

**Keywords:** phenolic compounds, carotenoids, vitamin C, arecaceae, TEAC, ORAC

## Abstract

Jeriva (*Syagrus romanzoffiana*) is a fruit from palm trees of the Arecaceae family, widely distributed in tropical and subtropical areas of Latin America. It has low production costs and high productivity throughout the year; however, its consumption is very low, and the production goes almost entirely to feed animals or to waste. To improve its consumption, a good characterization of the whole fruit is necessary. The objective of this work was to evaluate the jeriva pulp, peel and seeds according to carotenoids, phenolic compounds, vitamin C, tocopherols and antioxidant potential using HPLC, microplate readers and spectrophotometric methods. Every part of the fruit exhibited antioxidant capacity in the ORAC and TEAC tests, which can be attributed to its high concentration of polyphenols. Carotenoids were more present in the pulp and peel and almost absent in the seeds. Vitamin C ranged from 12 ± 1 for the seeds up to 92 ± 3 mg/100 g for the pulp. The total phenolic content was quantified between 473 ± 39 for the seeds and 1089 ± 32 mg of gallic acid equivalents (GAEs)/100 g for the pulp. These results demonstrate that all parts of this fruit have important bioactive nutrients, with promising perspectives for further scientific approaches and for composing formulations of food products to enhance functional properties.

## 1. Introduction

Vegetable compounds have been traditionally used in various fields such as the food, cosmetic, medical and chemical industries [1,2,3,4,5]. South America is well known for its extensive biodiversity in fruits and vegetables, with many of them not well known, researched, or consumed. The World Health Organization (WHO) recommends a diet based on the consumption of different fruits and vegetables each day, but this recommendation is hardly followed around the globe, where people respect only the diversity across food groups [6]. Most fruits are known as good sources for several compounds that are good for our health, such as minerals, vitamins and phenolic compounds that can be beneficial in several ways [7,8,9]. These compounds can be used as functional ingredients in the food industry, as natural conservatives or colorants or simply by adding their functional properties to the food. The knowledge and use of wild fruits in human nutrition could be a good source for public policies against malnutrition.

**Figure 1:**
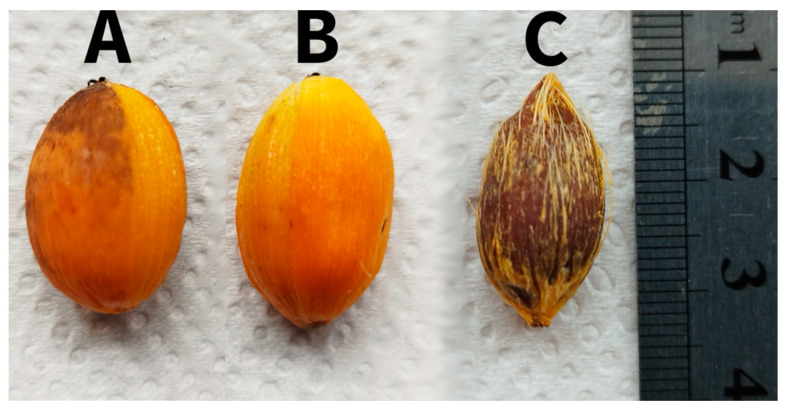
Whole jeriva fruit peeled on its right side (**A**), completely peeled fruit (**B**) and seed (**C**).

Jeriva (*Syagrus romanzoffiana*) is the Brazilian common name of an orange, round-to-oval-shaped fruit with approximately 2 cm of diameter (Figure 1) from a palm tree from the *Arecaceae* family, which is widely distributed in South America, considering this palm tree grows well in tropical and subtropical climates [10,11,12]. Whereas there are reports of regional consumption of these fruits, there are no plantations of these palms, and their fruit must be collected from the wild, being part of the diet of only a few regions and almost totally unknown to people from big consumer centers [13].

The fruit can be divided into three major parts: the pulp (47%), the seed (43%) and the peel (10%). The peel is very thin, orange and hard to separate, so it is normally consumed with the pulp. The pulp is also orange, very sweet and fibrous, with approximately 13% of insoluble fibers, 3% of proteins, 0.45% of lipids, 30% of carbohydrates and 44% of moisture. The seed is a huge part of the fruit composed approximately of 18% proteins, 21% lipids, 12% carbohydrates and 36% insoluble fibers [14].

The fruits from the *Arecaceae* family (among them, *Syagrus romanzoffiana, Attalea phalerata* and *Mauritia flexuosa)* have been attracting the attention of several studies focused mainly on their potential properties. They have been reported as fruits rich in their mineral composition, good fatty acids and antioxidant activities, which can be attributed to the higher amounts of polyphenols and carotenoids found in those fruits [5,9,10].

Several recent studies have focused on the use of jeriva seeds to produce an alternative biodiesel [11,12]. The results have been promising, but to produce the biodiesel, the fruit must be depulped, and the pulp and the peel are wasted.

The studies that report the higher concentration of nutraceutical compounds in the fruit are mainly focused on its sweet and fibrous pulp. Only a few studies are interested in the seeds, but mainly for their oil for biodiesel purposes. It was not possible to find a study that separated the three main parts of the fruit to characterize them separately to maximize their potential consumption. Therefore, the objective of this work was to characterize the pulp, peel and seeds of jeriva regarding the composition of carotenoids, phenolic compounds and antioxidants.

## 2. Materials and Methods

### 2.1. Plant Material

All fruits were collected in Sao Paulo (Brazil) State between May and July 2023. They were collected at harvest maturity and selected according to the absence of damage. They were washed and then sanitized with an aqueous solution of 0.66% (*v*/*v*) sodium hypochlorite (content of active chlorine: 3%). After the sanitation, the fruits were peeled, and the pulp was separated from the seed. The three different parts of interest of these fruits were dried in a vacuum oven at 35 °C until they reached constant weight. Each part was ground in a knife mill and stored in vacuum bags at 4 °C until further use.

### 2.2. Carotenoid Extraction

For this extraction, a method described previously in the literature [15] was used. Three different extracts were made: one for the pulp (500 mg), a second for the peel (200 mg) and a third for the seeds (1 g). In each one, the dry matter was dissolved in 5 mL of methanol/tetrahydrofuran (THF) (1/1, *v*/*v*) containing 0.1% BHT (to avoid oxidative degradation). 200 mg Magnesium oxide, 200 mg sodium sulfate and 100 µL solution of β-apo-8′-carotenal (internal standard) were added to each sample. The mixture was homogenized (cooled with ice) using an ultra turrax at 10,000 rpm (T25, IKA, Staufen, Germany) for 5 min. The supernatant was filtered under vacuum through filter paper no. 390 (Filtrak, Niederschlag, Germany) on a Buchner funnel. The extraction of the remaining residue was repeated until the residue became colorless. The combined supernatants were concentrated in a rotary evaporator at 30 °C with reduced pressure (ca. 20 Pa). Using an ultrasonic bath, the residue was re-dissolved using methanol/THF (1/1, *v*/*v*) containing 0.1% BHT until the solution reached a final volume of 5 mL. The pulp extract was prepared at a concentration of 100 mg/mL, the peel extract at 40 mg/mL and the seed extract at 200 mg/mL. The extracts were centrifuged for 5 min at 18,500× *g*, and the supernatants were used for carotenoid HPLC analysis.

### 2.3. Carotenoid Identification and Quantification

The identification of carotenoids was performed by comparing the spectral data in the samples with external standards (β-carotene, α- and β-cryptoxanthin, lutein, lycopene, rubixanthin, violaxanthin and zeaxanthin) [16]. An analysis was performed by HPLC with a diode array detector at 450 nm (Merck Hitachi, Darmstadt, Germany). The chromatographic separation was performed on a Develosil RP-aqueous (250 mm × 4.6 mm, 5 µm) C30 column (Phenomenex, Aschaffenburg, Germany) and a precolumn (C18: 4.0 × 3.0 mm, 5 μm) at 13 ± 1 °C using the following gradient: solvent A (MeOH) and solvent B (MtBE): 0 min (90:10), 40 min (50:50); 42 min (40:60), 65 min (40:60), 70 min (90:10) and 75 min (90:10). The flow rate was 1 mL/min. Carotenoid contents were quantified by a 5-point calibration curve of external standards. The identifications were performed by comparison with external standards according to their retention times, DAD absorbance spectra and mass spectra. The results are expressed as mg of carotenoid per 100 g of dry matter.

### 2.4. Sample Extraction for HPLC Phenolic Compound Determination

Samples of ground and homogenized pulp, seed and peel powder were weighed separately in three different 50 mL polypropylene tubes (all samples in triplicate) and extracted according to a method described in the literature [17], with slight modifications. In each tube, 3 mL of hydrochloric acid (1 M) was added and then agitated in a Vortex shaker for 30 s. After that, the samples were left in a water bath for 30 min at 37 °C. After this time, 3 mL of sodium hydroxide (2 M) was added, mixed in the Vortex for 30 s and left again in the water bath for 30 min at 37 °C. In the next step, metaphosphoric acid (0.75 M) was added. Each resulting solution was vortexed for 30 s and centrifuged for 5 min at 3500 g. The supernatant of each one was recovered and stored in brown round flasks.

The precipitate was subjected to an ethanol/water (70/30) extraction. A total of 20 mL of ethanol was added to the residue and left in an ultrasonic bath for 20 min at 40 °C. After that, the sample was centrifuged, and the supernatant was recovered together with the previous one. This extraction was repeated twice, reducing the ultrasonic bath time to 10 min, and in the last repetition, the amount of ethanol was reduced to 10 mL.

The extract collected in the brown round flask was dried in a rotary evaporator at 35 °C, and the final residue was collected with 5 mL of methanol/water (70/30). The extracts were prepared with a concentration of 100 mg/mL for the pulp and the peel and 200 mg/mL for the seeds. The sample was centrifuged (5 min, 18,500× *g*) prior to the HPLC analysis.

### 2.5. HPLC Analysis for Polyphenols

The HPLC analysis for polyphenols was made using a photodiode array detector (L-7450A, Merck Hitachi, Darmstadt, Germany), using a method described in the literature [17]. A reversed-phase Luna C18 column (250 mm × 4.6 mm, 5 µm, Phenomenex, Aschaffenburg, Germany) with the D-7000 interface (Merck Hitachi) was used. A total of 50 µL of each sample was injected into the system. The mobile phase was a gradient of hydrochloric acid 0.5% (*v*/*v*) in water (solution A) and methanol (solution B). The flow rate was 0.8 mL/min for 160 min using the following gradient: 0–2 min 0% B isocratic; 2–6 min linear gradient 0–15% B; 6–12 min, 15% B isocratic; 12–17 min linear gradient 15–20% B; 17–35 min, 20% B isocratic; 35–90 min linear gradient 20–35% B; 90–132 min 35% B isocratic; 132–150 min linear gradient 35–80% B; 150–160 min linear gradient 80–0% B. The results are expressed as mg of phenolic compound per gram of dry matter.

### 2.6. Vitamin C Determination

The vitamin C contents were determined using a method described in the literature [17]. Approximately 500 mg of peel and pulp and 1000 mg of seeds were weighted separately in three different Falcon tubes with 5 mL of H_3_PO_4_. Each one was mixed for 1 min in a Vortex shaker and then centrifuged for 5 min at 3500× *g*. After that, the supernatant was quantitatively transferred to a 10 mL volumetric flask that had its volume completed with H_3_PO_4_, resulting in extracts with 100 mg/mL for the pulp and the peel and 200 mg/mL for the seeds.

For the analysis, 200 µL of the corresponding calibration solution (ascorbic acid 1–100 µg/mL) or 200 µL of sample solution or 200 µL of distilled H_2_O (blank value) was added to a 1.5 mL Eppendorf tube. Then, 300 μL of TCA (5 g/100 mL) was added and mixed on the Eppendorf shaker for 5 min, and after that, they were centrifuged at 18,500× *g* for 5 min. A total of 300 μL of the supernatant was added to 100 μL of DNP reagent in a new reaction vessel and mixed. The mixture was tempered for 1 h at 60 °C in a thermomixer. The reaction vessels were then cooled in an ice bath for 5 min. After that, 400 μL of sulfuric acid (9 N) was added and mixed vigorously. The solutions were placed in the dark for 20 min and then measured spectrophotometrically at 520 nm. The results are expressed as mg of vitamin C per 100 g of dry matter.

### 2.7. Extraction for Total Phenolic Determination and Antioxidant Tests

For pulp and peel, approximately 0.5 g of each was weighted in different Falcon tubes with 5 mL of HPLC water. Each tube was mixed in a Vortex shaker and then centrifuged for 5 min at 3500× *g*. The supernatant was collected in a 20 mL volumetric flask, and the residue was extracted again with water. This process was repeated four times, resulting in an extract of 25 mg/mL.

For the seeds, a different extraction had to be made. Approximately 0.5 g of seeds was weighted in Falcon tubes with 1 mL of HCl (1.0 mol/L). After shaking for 1 min in a Vortex shaker, the mixture was kept for 30 min in a water bath at 37 °C. After that, 1 mL of NaOH (2.0 mol/L in 75% MeOH) was added, mixed and left in the water bath for another 30 min. Then, 1 mL of metaphosphoric acid (0.75 mL/L) was added, and after mixing, it was centrifuged for 5 min at 3500× *g*. The supernatant was collected in a 25 mL volumetric flask. The residue was washed with a water/methanol solution (1:1 *v*/*v*) and centrifuged, and the supernatant was collected. This procedure was repeated twice. The volume was filled with a water/methanol solution (1:1 *v*/*v*), resulting in an extract of 20 mg/mL.

### 2.8. Total Phenolic Determination

The total phenolic determination was made using the Folin–Ciocalteu method [18], with some modifications [19]. For each sample solution, 30 µL was poured into a 96-well microplate (Kisker Biotech, Steinfurt, Germany). The blank was made using 30 µL of water, and the standard curve was constructed using 30 µL of gallic acid (50–1000 µmol/L) instead of the samples. In each vial, 150 µL of Folin–Ciocalteu reagent (1:10) and 120 µL of sodium carbonate solution (0.7 mol/L) were added. After 2 h in darkness and at room temperature, the absorbance was measured at 740 nm and 30 °C using a microplate reader (FluoStar Optima (BMG Labtech, Offenburg, Germany)). The results were compared with the gallic acid standard curve and are expressed as gallic acid equivalents (GAEs) in mg/100 g of dry matter.

### 2.9. Trolox Equivalent Antioxidant Capacity (TEAC)

There is no official antioxidant capacity method for food products. Therefore, it is recommended that the analysis is made with various oxidation conditions and methods. The TEAC method is the most popular method based on electron transfer, which measures the capacity of an antioxidant to reduce an oxidant, changing its color, which can be correlated with the antioxidant potential of the sample [20].

The TEAC analysis was made using the method described in a previous study [21], with some modifications [19]. A total of 20 µL of the sample extracted for antioxidant tests was added to a 96-well microplate (for the blank, 20 µL of water was used, and for the standard curve, 20 µL of Trolox in a concentration range of 12.5 to 250 µmol/L). An ABTS working solution was prepared daily by diluting the ABTS stock solution with phosphate buffer (pH 7.4) to an absorbance of approximately 0.70 at 730 nm. In each well, 200 µL of ABTS solution was added, and the absorbance was measured at 30 °C. The results were compared with the Trolox standard curve and are expressed as Trolox equivalents (TEs) in mmol/100 g of dry matter.

### 2.10. Oxygen Radical Absorbance Capacity (ORAC)

The ORAC test is based on hydrogen atom transfer, which consists in measuring the decrease in the fluorescence of the protein fluorescein due to oxidative damage. Therefore, this method measures the ability of the sample’s antioxidants to protect this protein from oxidation [20].

This analysis was made using the method described in previous studies [19,22]. A total of 10 µL of water (blank) or Trolox (0.1–2.0 mmol/L) or the sample extracted for antioxidant tests was added to a 96-well microplate. Then, 25 µL of fluorescein (0.04 mg/L fluorescein in phosphate buffer pH 7.4) and 100 µL of phosphate buffer pH 7.4 were added to each well. The mixture was kept at 37 °C for 10 min. After that, 150 µL of AAPH solution was added to each well, and then the antioxidant measurement was made at 37 °C for 240 cycles of 1 min each. An excitation wavelength of 490 nm and an emission wavelength of 520 nm were applied in the assay. Wells for controlling the photostability of fluorescein were filled with phosphate buffer in place of the samples. To calculate the ORAC value of the samples, the relative fluorescence values at each minute were generated, and the area under the curve was calculated for the cycle. The final ORAC value is expressed as Trolox equivalents (TEs) in mmol/100 g of dry matter.

### 2.11. Statistical Analysis

The results are expressed as mean ± standard deviation (SD). One-way ANOVA followed by the Tukey test was performed using Microsoft Excel 365 and the statistical package XLMiner Analysis ToolPak (Frontline Systems, Incline Village, NV, USA) to evaluate possible significant differences between the results at *p* < 0.05. Pearson correlation analysis was conducted using Microsoft Excel 365.

## 3. Results and Discussion

The overall results obtained for vitamin C, total (poly)phenols and antioxidant capacities for pulp, peel and seeds are presented in Table 1.

Vitamin C contents ranged from 12.0 mg/100 g in the seeds to 92.3 mg/100 g in the pulp. With these results, the jeriva seeds could be compared to the fruit of persimmon [23], and the peel and the pulp could be compared with the values found in blackberries [19]. Vitamin C can be considered an essential compound in the human diet since it is not synthesized by humans. It is necessary for the biosynthesis of several compounds in the human body, is involved in protein metabolism and is also related to the prevention of certain diseases thanks to its antioxidative properties. Analyzing these data, jeriva fruit can be considered a good alternative for vitamin C consumption, being able to help to achieve the recommended dietary allowances (RDA) of 75 mg of vitamin C a day for human females and 90 mg for males [24].

The analysis of total phenolics by the Folin–Ciocalteu method resulted in contents ranging from 473 to 1089 mg GAEs/100 g, with the best result reported for the pulp and the lowest amount for the seeds. In this way, seeds can be compared in terms of their phenolic concentration (100–500 mg GAEs/100 g) to intermediate-phenolic-concentration fruits such as plums and guavas, while the peel and pulp can be compared to high-phenolic-level fruits such as the banana passion fruit [25]. These results were like those found in the literature [10], which tested the phenolic composition of jeriva pulp (JP) and kernel cakes (JCs) and obtained values of 851 mg GAEs/100 g for JP and 266 mg GAEs/100 g for JCs. In that study, the samples of the pulp and kernel cakes were submitted to a different preparation method. The samples were liquefied, frozen and dehydrated in a lyophilizer. The difference between the sample preparations may result in differences among the results.

Some of the polyphenols could be identified and quantified by HPLC (Table 2 and Figure S1). Hydroxy benzoic acid, procyanidin B2, coumaric acid and ferulic acid were found in all the samples, while fumaric acid was found only in the pulp and the peel. Catechin was found in the pulp and the seeds. These results are in accordance with the literature [26], which found hydroxy benzoic acid, caffeic acid, syringic acid, coumaric acid, ferulic acid and sinapic acid in jeriva pulp.

Throughout the study of polyphenols in jeriva in its soluble, insoluble and esterified portions, a study in the literature [27] reported the presence of several polyphenols such as O-methyl-gallic acid, monogalloyl hexoside, protocatechuic acid, p-hydroxybenzoic acid, ferulic acid, hydroxygallic acid, p-coumaric acid and sinapic-O-glucoside. The total amount of each compound varied according to the extraction method. In general, the free portion summed up a total of 157.5 µg/g, the esterified portion 94.9 µg/g and the insoluble-bound portion 139 µg/g of phenolic compounds in the jeriva pomace. In the seeds, the values were much higher for the insoluble-bound extract (1082 µg/g), and the esterified portion was similar (93.9 µg/g), while the soluble one was lower (52.4 µg/g) than the one found in the pomace.

The values obtained for antioxidant capacity were higher for the ORAC method. The pulp had higher values in both methods (16.9 mmol TEs/100 g in ORAC and 3.5 mmol TEs/100 g in TEAC), while the seeds had the lowest (10.9 and 1.7 mmol TEs/100 g, respectively, for ORAC and TEAC). The literature for jeriva indicates an antioxidant capacity for the pulp that is almost twice the value of the kernel cake using the ABTS method and almost triple when using the DPPH method [10]. According to the literature, the values of antioxidant capacity in the ORAC method are higher than those of well-known fruits such as melon [28], tomatoes [29] and orange juice [20]. The jeriva pulp can be compared to concentrated lingonberry, and the jeriva seeds can be compared to pomegranate according to the antioxidant capacity measured by the TEAC analysis [20].

**Figure 2:**
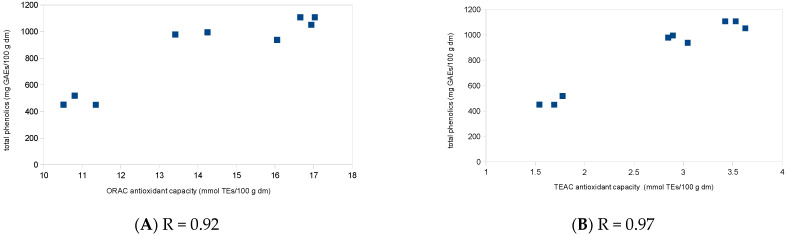
Pearson correlations (*p* < 0.01) of antioxidant capacities of jeriva samples. (**A**) ORAC and (**B**) TEAC analyses; dm: dry matter; ORAC: oxygen radical absorbance capacity; TEAC: Trolox equivalent antioxidant capacity; TEs: Trolox equivalents.

Polyphenols are often associated with the prevention of diseases and health promotion, especially because these compounds are normally correlated to their antioxidant capacity [30,31,32,33]. This correlation was found in the results of this work. While the pulp, with higher amounts of polyphenols, presented a higher antioxidant capacity, the seeds, with a lower polyphenol content, showed a lower antioxidant capacity (Figure 2).

**Figure 3:**
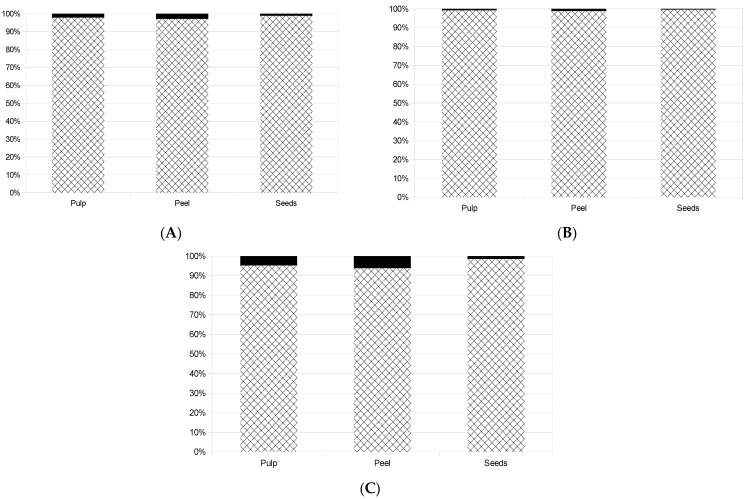
Portions (%) of total phenolics (**A**), ORAC antioxidant capacity (**B**) and TEAC antioxidant capacity (**C**) in jeriva samples caused by vitamin C (⬛).

Vitamin C is well known for its antioxidant properties in fruits, but it is commonly less stable than polyphenols [34,35]. The contributions of vitamin C and polyphenols to total antioxidant activity can differ extensively among fruits [36]. Some authors found that vitamin C was responsible for only 0.35% of the antioxidant activity of red grapes, reaching 8.6% in grapefruit [37]. In citrus fruits, the effect is much more relevant (around 80%) than in non-citrus ones (around 1–5%) [38]. To investigate the effect of vitamin C on the results of total phenolic contents, the ORAC and TEAC analyses were conducted again using ascorbic acid in different concentrations as the sample. After that, using the results of vitamin C reported in Table 1, it was possible to determine the interference of vitamin C in the results of the fruit. The portion not related to vitamin C can be associated with other main compounds, such as polyphenols. Figure 3 shows the effect of vitamin C on the analysis of antioxidant capacities and total polyphenols. The tendencies among the samples were nearly the same across the tests. The analysis of the peel was more affected by vitamin C, followed by the pulp and the seeds. The highest effect of vitamin C could be seen in the peel analysis using the TEAC test, where 6.2% of the result could be explained by vitamin C (followed by the pulp with 4.7% and the seeds with 1.4%). The total phenolic analysis had an effect of 2.9%, 2.2% and 1.1% for the peel, pulp and seeds, respectively, while vitamin C was responsible for 1.2, 0.8 and 0.3% of antioxidant capacity for the peel, pulp and seeds, respectively, in the ORAC test.

In another study, vitamin C affected the TEAC analysis in a range of 10% (concentrated lingonberry) to 70% (acerola puree) and affected the ORAC test in a range of almost 10% (cranberry puree) to 80% (acerola puree) [20]. The low values found for the jeriva pulp, peel and seeds, added to the high correlation coefficient presented in Figure 1, can be evidence that in this fruit, the phenolic compounds are responsible for the antioxidant capacity.

Table 3 shows the five carotenoids identified by HPLC analysis in the pulp, peel and seeds of jeriva: (*all-E*)-β-carotene, (*9Z*)-β-carotene, (*13Z*)-β-carotene, (*all-E*)-lutein and (*all-E*)-β-cryptoxanthin. The chromatograms of the carotenoids are presented in Figure S2. The compounds (*all-E*)-β-Carotene, (*9Z*)-β-carotene and (*all-E*)-lutein were found in higher quantities in the peel than in the pulp, suggesting the beneficial effect of the consumption of this part of the fruit. The amount of (*all-E*)-lutein found in the pulp can be compared to that of fresh apricots and in the peel to that of fresh peppers [39], while the amount of (*all-E*)-β-carotene in the peel can be compared to that of papaya fruit [40].

Jeriva pulp and cake residue are also known for their prebiotic potential, which promotes better growth of probiotic strains compared to commercial FOS [10]. Furthermore, there are reports about the presence of α-tocopherol in the fresh pulp of jeriva [26]. In the present work, it was not possible to identify any tocopherol in the dried samples using extraction with or without saponification. Hence, further studies using the fresh fruit as samples for the analysis are recommended. Despite the very promising potential of the fruit, a cytotoxicity test should be carried out in future studies to verify the possibility of its safe use in foods and by-products.

## 4. Conclusions

The pulp, peel and seeds of jeriva contain several compounds that can be beneficial for human health. Although the fruit does not have vitamin C and carotenoids among the highest sources of its contents, it can be considered a good source of bioactive compounds in general. The synergistic effect of all the compounds found in the fruit could be positive and stimulate fruit consumption and research. The knowledge of all these properties can be an incentive to commercial use of the fruit, as well as its use as an ingredient in several formulations in the food, cosmetic and pharmaceutical industries, increasing the functional properties of the formulations.

## Figures and Tables

**Table 1:** Contents of vitamin C, total phenolics and antioxidant capacities of pulp, peel and seeds from jeriva.

	Vitamin C (mg/100 g dm)	Total Phenolics (mg GAEs/100 g dm)	ORAC (mmol TEs/100 g dm)	TEAC (mmol TEs/100 g dm)
Pulp	92.3 ± 3.5 ^a^	1089 ± 32 ^a^	16.9 ± 0.2 ^a^	3.5 ± 0.1 ^a^
Peel	81.7 ± 2.3 ^b^	971 ± 30 ^b^	14.6 ± 1.4 ^b^	2.9 ± 0.1 ^b^
Seeds	12.0 ± 1.0 ^c^	473 ± 39 ^c^	10.9 ± 0.4 ^c^	1.7 ± 0.1 ^c^

The values are the means of triplicate determinations ± standard deviation. Values with different superscript letters within one column are significantly different (*p* < 0.05); dm: dry matter; GAEs: gallic acid equivalents; ORAC: oxygen radical absorbance capacity; TEs: Trolox equivalents; TEAC: Trolox equivalent antioxidant capacity.

**Table 2:** Contents of identified polyphenols (HPLC) in pulp, peel and seeds from jeriva.

mg/g dm	Fumaric Acid	Catechin	Hydroxy Benzoic Acid	Procyanidin B2	Coumaric Acid	Ferulic Acid
Pulp	0.45 ± 0.18	0.89 ± 0.17	0.15 ± 0.02	0.06 ± 0.01	0.05 ± 0.01	0.20 ± 0.04
Peel	0.35 ± 0.13	-	0.08 ± 0.01	0.09 ± 0.01	0.10 ± 0.01	0.15 ± 0.08
Seeds	-	0.23 ± 0.06	0.10 ± 0.02	0.04 ± 0.00	0.01 ± 0.00	0.07 ± 0.04

dm: dry matter.

**Table 3:** Contents of major carotenoid compounds (mg/100 g dm) in pulp, peel and seeds of jeriva.

mg/100 g dm	(*all-E*)-β-Carotene	(*9Z*)-β-Carotene	(*13Z*)-β-Carotene	(*all-E*)-Lutein	(*all-E*)-β-Cryptoxanthin
Pulp	3.14 ± 0.08	0.23 ± 0.04	0.72 ± 0.03	0.11 ± 0.01	0.03 ± 0.00
Peel	5.16 ± 0.33	0.32 ± 0.04	0.70 ± 0.08	0.56 ± 0.01	-
Seeds	0.63 ± 0.07	0.03 ± 0.00	0.13 ± 0.01	-	0.02 ± 0.01

dm: dry matter.

## Data Availability

Data are contained within the article.

## Supplementary Materials

The following supporting information can be downloaded at https://www.mdpi.com/article/10.3390/antiox13060711/s1: Figure S1. Chromatograms of polyphenols in jeriva pulp (A), peel (B) and seeds (C). The phenolic compounds quantified were fumaric acid (1), catechin (2), hydroxy benzoic acid (3), procyanidin B2 (4), coumaric acid (5) and ferulic acid (6); Figure S2. Chromatograms of carotenoids in jeriva pulp (A), peel (B) and seeds (C). The carotenoids quantified were (*all-E*)-lutein (1), (*all-E*)-β-cryptoxanthin (2), (*13Z*)-β-carotene (3), (*all-E*)-β-carotene (4) and (*9Z*)-β-carotene (5). (IS) represents the internal standard β-apo-8′-carotenal.
