# Supplementary material for: Bioactive Compounds and Antioxidant Capacity of Pulp, Peel and Seeds from Jeriva (Syagrus romanzoffiana)
Source: Antioxidants (Basel). 2024 Jun 12;13(6):711. doi: 10.3390/antiox13060711 (PMC11200598; doi:10.3390/antiox13060711)
Supplement: Supplementary file 1 [file antioxidants-13-00711-s001.zip › antioxidants-3034444-supplementary.pdf]

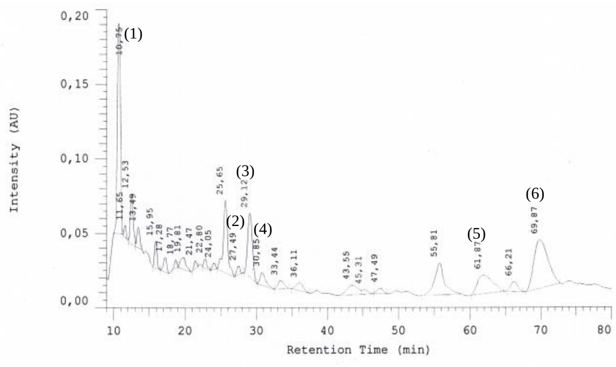

(A)

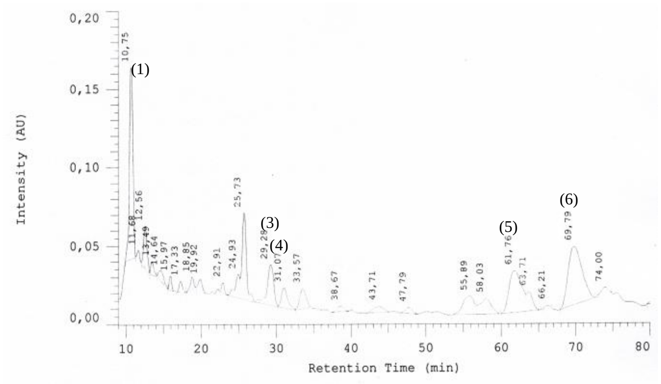

(B)

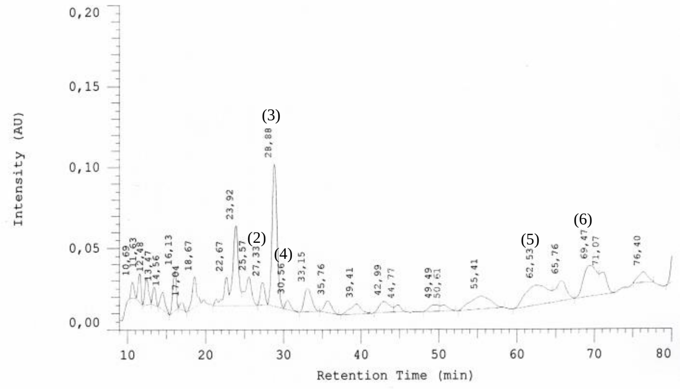

(C)

Figure S1. Chromatograms of polyphenols of jeriva pulp **(A)**, peel **(B)** and seeds **(C)**. The phenolic compounds quantified were: fumaric acid **(1)**, catechin **(2)**, hydroxy benzoic acid **(3)**, procyanidin B2 **(4)**, coumaric acid **(5)** and ferulic acid **(6)**.

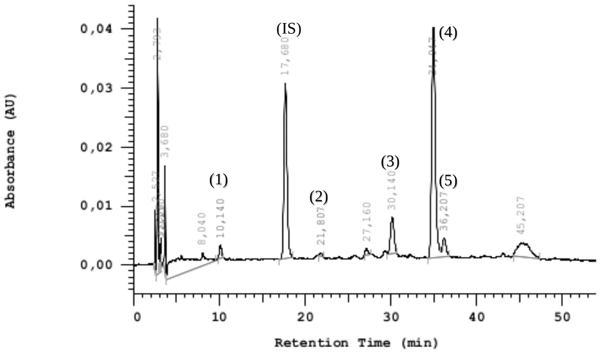

(A)

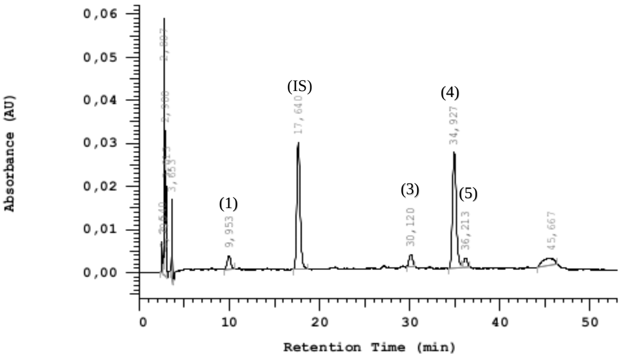

(B)

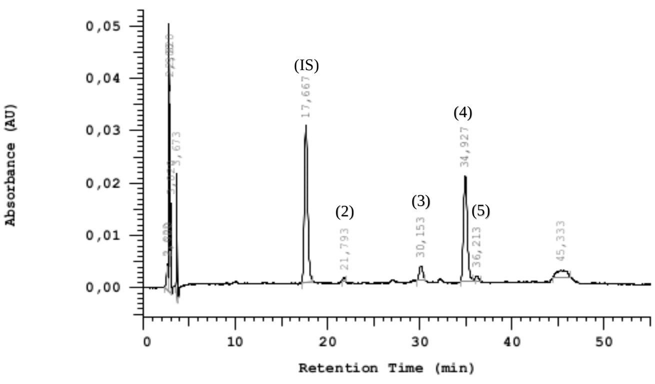

(C)

Figure S2. Chromatograms of carotenoids of jeriva pulp **(A)**, peel **(B)** and seeds **(C)**. The carotenoids quantified were: (*all-E*)-lutein **(1)**, (*all-E*)-β-cryptoxanthin **(2)**, (*13Z*)-β-carotene **(3)**, (*all-E*)-β-carotene **(4)** and (*9Z*)-β-carotene **(5)**. **(IS)** represents the internal standard β-apo-8′-carotenal.
